# Impact of Switch Options on the Economics of Pneumococcal Conjugate Vaccine (PCV) Introduction in Indonesia

**DOI:** 10.3390/vaccines8020233

**Published:** 2020-05-18

**Authors:** Auliya A. Suwantika, Neily Zakiyah, Arif S. W. Kusuma, Rizky Abdulah, Maarten J. Postma

**Affiliations:** 1 Department of Pharmacology and Clinical Pharmacy, Faculty of Pharmacy, Universitas Padjadjaran, Bandung 40132, Indonesia; neily.zakiyah@unpad.ac.id (N.Z.); r.abdulah@unpad.ac.id (R.A.); 2 Center of Excellence in Higher Education for Pharmaceutical Care Innovation, Universitas Padjadjaran, Bandung 40132, Indonesia; m.j.postma@rug.nl; 3 Center for Health Technology Assessment, Universitas Padjadjaran, Bandung 40132, Indonesia; 4 Department of Biological Pharmacy, Faculty of Pharmacy, Universitas Padjadjaran, Bandung 40132, Indonesia; arif.satria@unpad.ac.id; 5 Rutgers Graduate Program in Molecular Biosciences, Rutgers University, Piscataway, NJ 08854, USA; 6 Unit of Global Health, Department of Health Sciences, University Medical Center Groningen (UMCG), University of Groningen, 9713AV Groningen, The Netherlands; 7 Department of Economics, Econometrics and Finance, Faculty of Economics and Business, University of Groningen, 9747AE Groningen, The Netherlands; 8 Unit of Pharmaco-Therapy, Epidemiology and Economics (PTE2), Department of Pharmacy, University of Groningen, 9713AV Groningen, The Netherlands

**Keywords:** Gavi-AMC, potential saving, vaccine price, multi-dose, cold chain

## Abstract

As one of Gavi, the Vaccine Alliance (previously the Global Alliance for Vaccines and Immunization), graduating countries, Indonesia is still eligible to access Gavi price for PCV13, PCV10 A and B. This study aims to estimate the economic impact of switch from the existing product/presentation of PCV (single-dose of PCV13) to the new product/presentation of PCV (multi-dose of PCV13, PCV10 A and B) since PCV is one of the most expensive vaccines in the Expanded Program on Immunization (EPI) schedule. Assuming that Gavi-Advance Market Commitment (AMC) price for all PCVs can be accessed in 2021, the use of multi-dose PCV13, PCV10 A and PCV10 B with Gavi-AMC price in 2021–2024 were considered as respective scenarios. The result showed that the scenario assuming the use of single-dose of PCV13 with contract price in 2019–2020 that would be switched into multi-dose of PCV10 B with Gavi-AMC price in 2021–2024 resulted in the highest potential saving, compared with other scenarios. Our analysis suggests an economic advantage to switch from single-dose into a multi-dose presentation. Vaccination coverage, vaccine price, vaccine wastage and additional Gavi-AMC vaccine costs were considered to be the most influential parameter affecting the savings in all scenarios. Applying the effectiveness of PCV13 and PCV10 A on reducing the risk for invasive pneumococcal disease (IPD), potential averted incidence of IPD in children under one year of age during 2019–2024 would be 246,164 and 105,587 in both scenarios. Despite the result confirmed that PCV13 may provide an additional benefit, a more comprehensive economic evaluation study is required to investigate further the comparison of cost-effectiveness values among all PCVs in Indonesia.

## 1. Introduction

*Streptococcus pneumoniae* causes a variety of diseases, such as pneumonia, acute otitis media (AOM) and invasive pneumococcal disease (IPD) [1]. The World Health Organization (WHO) estimated that 5.8 million children under five years old die every year, with 300,000 being estimated to be caused by pneumococcal infections [2]. The incidence of IPD varies by country, which has been reported to be 5-416/100,000 in developing countries [3]. Approximately 75% of IPD cases occur in children under two years old [3]. In Indonesia, pneumococcal disease is the major cause of infant mortality [4]. The United Nations Children’s Fund (UNICEF) highlighted that 71 children in Indonesia are infected with pneumonia every hour [5]. To decrease morbidity and mortality rate of pneumonia in children under five years old, the government of Indonesia has tried to intensify prevention and control strategies, such as through the case management of pneumonia in the community, health centers and hospitals, exclusive breastfeeding for the first six months of life, improvement of nutrition and prevention of low birth weight [6]. Nevertheless, the number of outpatients, hospitalized and fatal cases due to pneumonia remains high. 

Infant vaccination is considered to be the most effective way to prevent infections and reduce the burden, mortality and sequelae in children (direct effect) and adult populations (indirect effect). Three pneumococcal conjugate vaccines (PCVs) have been licensed to immunize children against pneumococcal disease: (i) a 7-valent PCV (PCV7), which is no longer available; (ii) a 10-valent PCV (PCV10); and (iii) a 13-valent PCV (PCV13) [7]. PCV10 and PCV13 differ in the number of serotypes they contain, the amount of capsular polysaccharide antigen per serotype, the carrier proteins these antigens are conjugated to, and the method used for conjugation [8,9,10]. In particular, both PCV10 and PCV13 have proven to be effective in preventing pneumococcal diseases. The vaccine manufacturers recommend that infants who receive the first dose of one of the vaccines should complete the immunization course with the same vaccine [8,9]. Recently, the WHO has revisited this recommendation, now specifically mentioning that if this is not possible, the other PCV should be used to complete the regimen [2]. Several countries have switched from PCV13 to PCV10 (or vice versa) in their infant immunization programs [11,12,13,14,15,16,17,18]. Other countries have considered switching between PCVs as part of their immunization program based on local epidemiology and programmatic factors [2,19].

Epidemiological surveillance of *S. pneumoniae* serotype carriage in developing countries is important to implement appropriate vaccination strategies. A previous study by Hadinegoro et al. confirmed that serotypes 6A/B (22% of pneumococcal strains) were the most frequent serotypes found in Indonesia [20]. Another study by Dunne et al. mentioned that serotypes 15B/C, 23F, NT2 (a non-capsulated lineage), 19F and 6A were the most common serotypes identified [21]. Overall, approximately 46.3% and 32.3% of pneumococcal infections belonged to PCV13 and PCV10 serotypes, respectively [21]. Following the results of these studies, the government of Indonesia has initiated to introduce PCV13 in 2017 in two selected districts (West and East Lombok), which represented districts in a province with the highest percentage of pneumonia cases in children under five years old in Indonesia [4,22]. As one of Gavi, the Vaccine Alliance (previously the Global Alliance for Vaccines and Immunization), graduating countries, Indonesia is still eligible to access Gavi prices for both PCV10 and PCV13 [23]. According to Gavi’s latest report, there are five products/presentations of PCVs that can be procured via UNICEF Supply Division (SD) in 2020 [24]. These vaccines are single-dose (one dose/vial) and multi-dose (four doses/vial) PCV13, multi-dose (four doses/vial) PCV10 A, single-dose (one dose/vial) and multi-dose (five doses/vial) PCV10 B as the newest vaccine [9]. In comparison with PCV10 A (serotypes 1, 4, 5, 6B, 7F, 9V, 14, 18C, 19F and 23F) and B (serotypes 1, 5, 6A, 6B, 7F, 9V, 14, 19A, 19F and 23F), PCV13 contains three additional antigens (serotypes 3, 6A and 19A). More detailed information can be seen in Table 1. 

Up to now, there is no WHO position paper on a preference for a specific PCV-product, which allows countries to make decisions for a product based on local epidemiological and programmatic considerations. In the context of Indonesia, it is critical to estimate the economic impact of switch from existing product/presentation of PCV (single-dose of PCV13) to new product/presentations of PCV (multi-doses of PCV13, PCV10 A and B) as PCV is one of the most expensive vaccines in the Expanded Program on Immunization (EPI) schedule. Since PCV recommendations vary worldwide [14], this evidence is required to support vaccine introduction, optimize the impact of immunization and warrant the government’s investment in the immunization program that is considered to be of great public health value.

## 2. Interchangeability

PCVs have been used since 2000, with the licensure of PCV7. Currently, there are only two available PCVs: PCV10 and PCV 13. PCV introduction in lower- and middle-income countries (LICs and MICs, respectively) began in 2009 and has continued to increase over time. WHO has recommended that PCVs should be administered in infants using a 2primary(p)+1 or 3p+0 schedule, with the primary doses of each schedule administered at six months of age and the booster dose of the 2p+1 administered at nine months of age or later. Intervals between doses are generally at least eight weeks apart for the two primary doses in the 2p+1 schedule and at least four weeks apart for the 3p+0 schedule. Several published studies have confirmed vaccine-specific data on immunogenicity, safety, effectiveness and interchangeability, which demonstrated that both PCV 10 and PCV 13 exhibited grossly similar overall characteristics and impacts on the outcomes with some specific differences [11,14,28,29,30,31,32,33]. 

### 2.1. Immunogenicity 

Both PCV10 and PCV13 induce antibodies against the serotypes common across the two vaccines. Although there are small differences in antibody response between the two products for the individual serotypes, PCV10 and PCV13 have comparable, albeit not identical, immunogenicity. A recent systematic review and meta-analysis on associations between geographic region and immune response variations to PCVs showed a strong regional difference in the immune response [28]. Vaccinated individuals in Asia, Africa and Latin America were considered to have higher geometric mean concentrations (GMCs), compared to those in Europe and North America. Taking into account anti-pneumococcal antibodies, the result of the meta-analysis showed that GMCs in all PCVs were estimated to be 2.60 mg/mL (95% CI; 2.45–2.76 mg/mL), 2.30 mg/mL (95% CI; 2.21–2.39 mg/mL) and 1.60 mg/mL (95% CI; 1.54–1.65 mg/mL) after three doses of PCV7, PCV10 and PCV13, respectively [28]. Focusing on serotype 4 only, the study highlighted that PCV13 showed greater antibody responses than PCV7 and PCV10 [28]. Additionally, a systematic literature review on interchangeability between PCVs for pediatric use confirmed that four clinical trials showed that mixed schedules with a PCV10-to-PCV13 switch at boosting or a PCV13-to-PCV10 switch during priming or at boosting were similarly immunogenic with no apparent safety concerns [11].

### 2.2. Vaccine Safety 

Both PCV10 and PCV13 have strong safety profiles. Next to immunogenicity evaluation, a systematic review by Ciapponi et al. confirmed that there was no direct comparative information available on the interchangeability among PCVs, but they have similar immunogenicity and safety [14]. Another systematic review assessed the reactogenicity and safety of mixed schedules [11]. The study showed that switching from PCV13 to PCV 10 or vice versa at the time of boosting or from PCV13 to PCV10 during priming did not appear to raise safety concerns [11]. 

### 2.3. Effectiveness

Effectiveness of PCVs against IPD among children under five years of age in Africa can be used to explore the effectiveness of PCVs on IPD prevention in LICs and MICs [29]. The overall decline in IPD ranging from 31.7% to 80.1%. IPD caused by vaccine serotypes declined significantly at 35.0%–92.0%. A much higher decline (55.0%–89.0%) was found in children under two years of age. Of all vaccine serotypes, the relative proportions of serotypes 1, 5 and 19A doubled following vaccine roll-out [29]. In particular, a systematic review and meta-analysis of randomized controlled trials on the clinical effectiveness of PCVs confirmed that PCV13, PCV10 and PCV7 could reduce the risk for IPD with respective Odds Ratios (ORs) at 0.53 (95%CI; 0.36–0.78), 0.19 (95%CI; 0.11–0.33) and 0.37 (95%CI; 0.26–0.52) [30]. Focusing on the effectiveness of PCV13 against serotype 3 IPD, a study by Sings et al. showed the effectiveness of PCV13 at 63.5% (95% CI; 37.3%–89.7%) [31]. In addition, when PCV13 was compared with previous PCV7 immunization, the incidence rate ratio (IRR) of hospitalization for pneumonia in children was estimated to be 0.67 (95% CI; 0.62–0.74) [32]. Taking the cost-effectiveness into account, Ciapponi et al. highlighted that PCV10 and PCV13 were consistently more cost-effective than PCV7 [14]. Preliminary results of an economic evaluation study in Indonesia confirmed that PCV13 was considered to be more cost-effective than PCV10 [33]. 

## 3. Implementation of PCV Immunization in MICs

In 2012, the WHO has updated the recommendation about PCV introduction for children under two years old by giving prioritization to countries with high child mortality rates [34]. At this moment, the majority of people live in MICs, where the majority of vaccine-preventable diseases occur [35,36]. Despite the fact that the number of MICs introducing PCV is steadily increasing [37], MICs may be struggling with PCV implementation without external financial and technical support [38]. As a consequence, a comprehensive strategy is required to massively reduce the burden of mortality and morbidity due to pneumococcal disease in MICs within scarce budgets. To give an illustration about the uptake of PCV in lower- and upper-middle-income countries (LMICs and UMICs, respectively), a study by Tricarico et al. reported a significant difference in the uptake of PCV in LMICs (71%) and UMICs (48%), which was mainly caused by an unsuccessful process of MICs graduation [39]. It can be highlighted that once countries move from LICs into MICs or from LMICs into UMICs, financial assistance (e.g., Gavi’s support) are gradually phased out. In these cases, MICs are forced to explore the internal co-financing of vaccines. 

The WHO reported PCV as one of the most expensive new vaccines to be included in national immunization programs (NIPs) [34]. The price of PCV can vary over a wide range, from $3.30 per dose (when purchased through Gavi) up to $159.58 per dose (the price of PCV13 in the private sector) [40]. In order to fully fund their NIPs, MICs should expand their fiscal space to introduce PCV, supported with other activities, such as advocacy, technical assistance and training. As one of the MICs with approximately 270 million population [41], Indonesia has not yet added PCV to its NIP.

To give an illustration of the situation in other MICs with high numbers of the population, the Indian government has announced the possible introduction of PCV in a phased manner by 2017 [42]. In addition, the Chinese government has not yet included PCV in NIPs, but it is available at immunization clinics for a fee [43]. Although substantial achievements have been made with other immunization programs in MICs [44], the introduction of PCV and other new or under-used vaccines (e.g., rotavirus, human papillomavirus and Japanese encephalitis vaccine) in these countries are still lagging [45]. This situation might lead in at least 20% of the total population in MICs remaining unprotected from these important pathogens, which corresponds with a missed opportunity to dramatically reduce avoidable morbidity and mortality in countries where the majority of people live and the majority of vaccine-preventable diseases occur [32,46]. Obviously, financial constraints reflect an important obstacle. 

## 4. Advance Market Commitment (AMC)

AMC is a legally binding commitment by donors to provide funds to subsidize the vaccine purchase as an effort to reduce morbidity and mortality in low-resource countries [47]. This mechanism was aimed to encourage the research and development of new vaccines, and accelerate the availability of new vaccines in those countries [48]. It has been envisaged that this mechanism was designed both for early-stage (e.g., malaria, HIV/AIDS and tuberculosis vaccines) and late-stage development (e.g., PCV, rotavirus and human papillomavirus vaccines). An AMC for vaccines was initiated by the Center for Global Development in April 2005, which intended to create incentives for commercial investments in research, development and manufacturing of new vaccines [49]. 

An AMC pilot for PCV was launched in June 2009 with a joint commitment of several countries, such as Canada, Italy, Norway, the Russian Federation, the UK and the Bill and Melinda Gates Foundation [50]. The World Bank led the pilot together with the UNICEF and the WHO by securing $1.5 billion in AMC funding for the purchase of PCVs [51]. The Gavi Alliance, a public–private partnership, was appointed as the AMC secretariat and has committed to supporting eligible countries to purchase PCV through the AMC mechanism [52]. These eligible countries were allowed to apply for subsidized PCVs, with a maximum vaccine price of $3.50 per dose [53]. To support this pilot, the International Finance Facility for Immunization (IFFIm) provided $41.58 million to immunize more than three million children and prevent more than 1.5 million deaths by 2020 [54]. Through the Accelerated Vaccine Introduction (AVI) program that was initiated in 2008, Gavi aimed to broaden and speed up access to PCVs [55]. In addition, the WHO has actively developed supporting programs to help MICs on introducing PCV, such as through the Global Action Plan for the Prevention and Control of Pneumonia and Diarrhoea (GAPPD) and the MIC Task Force, which aimed to reduce the number of deaths due to pneumonia to be less than three children per 1000 live births by 2025 [32,56]. Furthermore, the WHO Strategic Advisory Group of Experts on Immunization (SAGE) has noted a dramatic increase in the implementation of new and under-utilized vaccines could potentially decrease morbidity and mortality of vaccine-preventable diseases, including pneumococcal disease, as mentioned in the Global Vaccine Action Plan (GVAP) of 2011–2020 [57,58]. 

To strengthen the implementation of AMC, the Pan American Health Organization (PAHO) Revolving Fund launched initiatives to assist Latin American countries in negotiating a lower cost of PCV through bulk procurement and provide technical assistance on supply management, planning, procurement system, quality assurance, warehousing and distribution [59]. The Program for Appropriate Technology in Health (PATH) has collaborated with private- and public-sector partners to accelerate the introduction of PCVs by providing affordable vaccines for low-resource countries [60]. In addition, Médecins Sans Frontières (MSF) launched a global campaign in 2015 to slash the price of PCV in MICs to be a maximum of $5 per child so that more children can be protected [39]. 

## 5. Impact of Switch Options on the Economics of PCV Introduction in Indonesia

Top-down cost estimation was applied to estimate the economic impact of switch options on the introduction of PCV in Indonesia. This estimation was based on the cost of the current project in two provinces, Nusa Tenggara Barat (NTB) and Bangka Belitung, and the expert judgment of those involved in developing the cost estimation [22]. Applying this approach, the economic impact of switch options due to parameters impacting vaccine cost (e.g., required doses, vaccine presentation, indicative wastage rate, vaccine coverage, targeted area, safety stock, vaccine price and other additional costs) and parameters impacting cold chain cost (e.g., cold chain volume per dose and cold chain cost per cm^3^) were estimated and presented in a tabular format (see Table 2). 

This analysis was performed for the period of 2019–2024. Selected districts and provinces were analyzed to implement PCV vaccination in Indonesia during that period. Several factors (e.g., the incidence rate, coverage of routine immunization program and local government readiness) were considered to prioritize districts and provinces on extending the introduction of vaccination. In 2019, PCV was introduced in two provinces and five districts, as targeted by the Ministry of Health (MoH). In 2020 and 2021, the introduction is targeted in four and six more provinces, respectively. In 2022 and 2023, it would be expanded in eight and 11 more provinces, respectively. In the end, nationwide vaccination is targeted to be implemented in 2024 (see Table 3).

As the MoH has initiated to introduce PCV13 in 2017, we applied a situation that PCV13 would be used in 2019–2024 as the base-case scenario. Since there is a regulation barrier to access Gavi-AMC price through procurement via UNICEF SD, the use of single-dose PCV13 with the government contract price was applied in 2019–2020. We assumed that the Gavi-AMC price for all PCVs can be accessed in 2021, as the starting year. In this analysis, three further scenarios were specifically compared with the base-case scenario. In particular, the use of multi-dose PCV13, PCV10 A and PCV10 B with Gavi-AMC price in 2021–2024 were considered in respective scenarios (see Table 4).

Total required doses were calculated by considering targeted children under one year of age in accordance with the vaccination schedule of PCV (2, 3 and 12 months old), vaccination coverage of 91%, buffer stock of 5% and vaccine wastage of 5% (single-dose) and 7% (multi-dose) [23,25,26]. The economic impact of switch options was analyzed by taking into account the potential savings on vaccine and cold chain costs from all scenarios, which were compared with the base-case scenario. In this analysis, we applied the government contract price of $20.83 for the use of single-dose PCV13 that was implemented in 2019–2020 [22]. For the Gavi-AMC vaccine, we estimated that there would be additional costs of forwarder (2.5%), distribution (12%) and insurance (5%) [22]. In addition, the UNICEF handling fee for new and under-used vaccines was estimated to be 3.5% for non-least developed countries [63]. Applying Gavi-AMC prices of $3.30, $2.90, $3.05 and $2.00 for single-dose PCV13, multi-dose PCV13, PCV10 A and PCV10 B, respectively [64], we estimated total vaccine cost in 2019–2024 would be $308.76 million; $292.22 million; $301.01 million and $239.50 million for the base-case and the respective scenarios of multi-dose PCV13, PCV10 A and PCV10 B. Considering cold chain cost of $0.0013 per cm^3^ [26,27], we estimated total cold chain cost in 2019–2024 would be $0.71 million; $0.33 million; $0.26 million and $0.32 million for the base-case and the respective scenarios. In comparison with the base-case, we estimated total savings in 2019–2024 would be $16.91 million, $8.20 million and $69.65 million for the respective scenarios of multi-dose PCV13, PCV10 A and PCV10 B (see Table 5).

**Figure 1:**
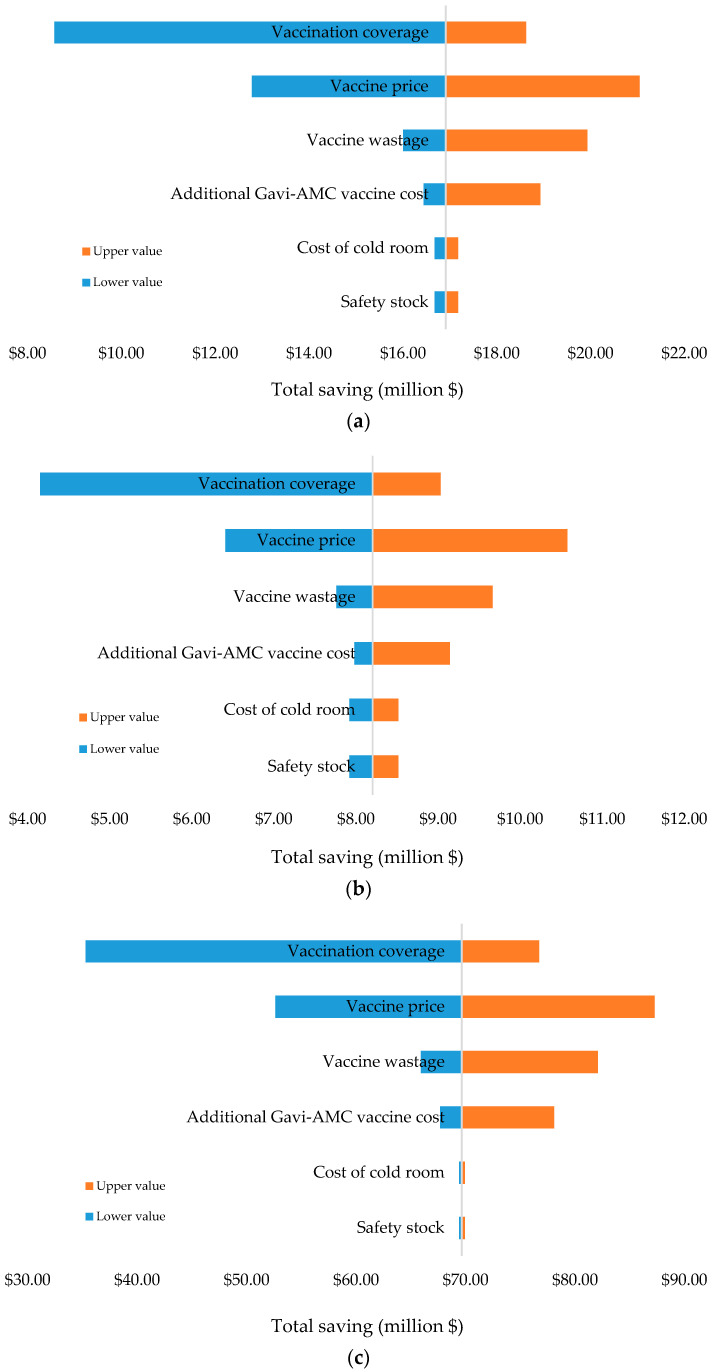
(**a**) Effect of different input parameters on total saving in the multi-dose PCV13 scenario. (**b**) Effect of different input parameters on total saving in the multi-dose PCV10 A scenario. (**c**) Effect of different input parameters on total saving in the multi-dose PCV10 B scenario.

**Figure 2:**
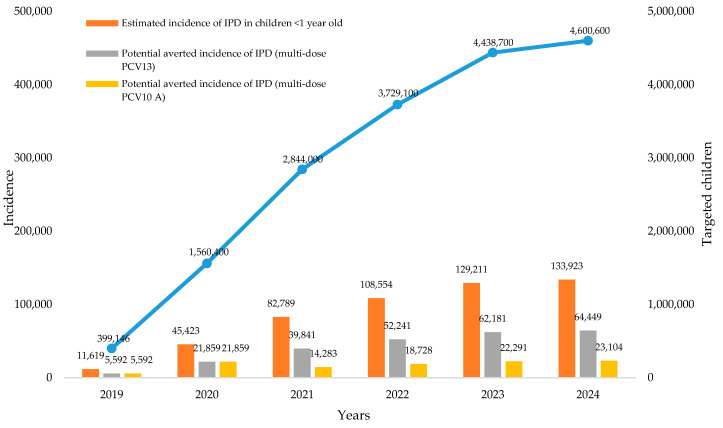
Potential averted incidence of invasive pneumococcal disease (IPD, children <1 years old) in targeted regions in scenarios for multi-dose PCV13 and PCV10 A.

Univariate sensitivity analyses were conducted to investigate the effects of the input parameters on the savings. The result showed that vaccination coverage, vaccine price, vaccine wastage and additional Gavi-AMC vaccine costs were considered to be the most influential parameters affecting the savings in all scenarios (see Figure 1). Applying the incidence rate of IPD in children under one year of age at 2911 per 100,000 population [62], and the effectiveness of PCV13 and PCV10 A on reducing the risk for IPD at ORs of 0.53 (95% CI; 0.36–0.78) and 0.19 (95% CI; 0.11–0.33), respectively [30], we estimated the potential averted incidence of IPD in children under one year of age during 2019–2024 at 246,164 and 105,587 for the respective scenarios of multi-dose PCV13 and PCV10 A (see Figure 2). Due to the lack of data on the effectiveness of PCV10 B, we could not take a potential averted incidence of IPD in children under one year of age into account for the last scenario.

## 6. Programmatic Considerations 

According to the WHO’s guideline on planning and managing vaccine introduction, several switch activities and other supporting activities should be taken into account, such as decision-making and switch request, planning and funding, vaccine procurement, vaccine distribution, human resources and training, document production and distribution, cold chain, logistics and vaccine management, surveillance and monitoring and evaluation [24,64].

In order to make a switch, several steps are required for evaluating the suitability of new product or presentation with support from advisory bodies (e.g., National Immunization Technical Advisory Group), developing a plan for the product/presentation switch (including activities, budget and timeline) and submitting the switch application to Gavi. With respect to planning and funding issues, the Ministry of Health (MoH) should establish a working group to coordinate switch activity in the country, develop risk management strategy for an adverse event following immunization (AEFI) and secure funding for activities not covered by Gavi or partners. To deal with vaccine procurement and distribution issues, the stakeholder should carry out inventory of existing product/presentation, schedule the consumption of old product/presentation before the introduction of new product/presentation, complete licensure of new vaccine products/presentations and arrange a distribution plan for new product/presentation to sub-national levels. In particular, training for health workers is required for dealing with the new vaccine product/presentation and the use of existing presentations ahead of switch. To support this training, there should be an updated document related to production and distribution (e.g., guidelines, handbook for health workers, frequently asked questions, fact sheets, video, posters, pre- and post-knowledge tests in local language) and reporting tools (e.g., vaccination logs, tools for wastage calculation) [64]. 

**Figure 3:**
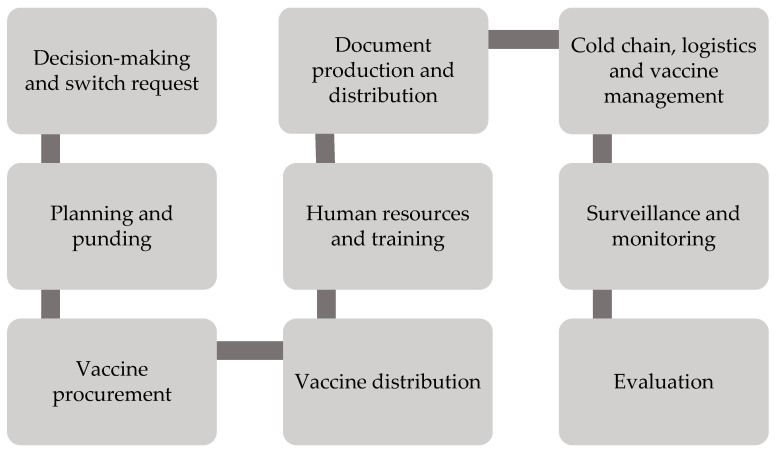
Planning guidance on product/presentation switch activity.

To manage product/presentation switch (including cold chain and logistics), updated stock management tools and more cold chain or dry storage space are critically required to include the new product/presentation in central and intermediary levels. In the context of surveillance and monitoring, the MoH should ensure adequate monitoring of vaccine wastage of the new product/presentation. In particular, the National AEFI Expert Review Committee should be able to provide technical guidance on real-world effectiveness causality assessment of AEFIs potentially related to the new product/presentation. To evaluate all related activities, there should be regular visits to local facilities to confirm switch activities, to correct the use of new products/presentations and to gather lessons on successes and challenges of the product/presentation switch (see Figure 3) [64]. 

## 7. Discussion

PCV, as one of new vaccines, is relatively expensive [65], and a 3p+0 schedule of PCV (at 2, 3 and 12 months of age) is applied for each child in Indonesia [22]. Therefore, it is critical to determine its optimal vial size, specifically in developing countries with limited immunization budget. Vial size refers to the size of the vial in which the vaccine is supplied. Multi-dose vials can have 4, 5, or 10 doses of vaccine in a vial while a single-dose vial has just one dose of the vaccine [26]. The manufacturing costs in a multi-dose vial are spread over many doses and therefore they tend to cost less per dose as compared to single-dose vials. Furthermore, multi-dose vials have lower cold chain costs. Hence, they are also thought to be associated with higher wastage [66,67]. This study highlights the economic value of switch options on the introduction of PCV in Indonesia. Despite the fact that multi-dose presentation has a higher indicative wastage rate (7%) than single-dose presentation (5%), the price of multi-dose presentation vaccines is much lower than single-dose presentation. Our analysis suggests an economic advantage to switch from single-dose into a multi-dose presentation. In terms of potential savings, the option of single-dose PCV13 with the contract price in 2019–2020 that would be switched into multi-dose PCV10 B with Gavi-AMC price in 2021–2024 resulted in the highest potential saving, compared with other scenarios. This situation was caused by the fact that a multi-dose of PCV10 B, which was targeted to be available by the end of 2020, had the lowest estimated price compared with other PCVs under Gavi-AMC price. Vaccination coverage, vaccine price, vaccine wastage and additional cost of the Gavi-AMC vaccine were considered to be the most influential parameter affecting the savings in all scenarios. In particular, we briefly estimated potential averted incidence of IPD in children under one year of age by taking into account the use of multi-dose PCV13 and PCV10 A, respectively. We estimated a potential averted incidence of IPD in children under one year of age during 2019–2024 would be 246,164 and 105,587 in both scenarios. Despite the result confirmed that PCV13 may provide an additional benefit, a more comprehensive economic evaluation study is required to investigate further the comparison of cost-effectiveness values among all PCVs in Indonesia.

This is the first study to analyze the economic impact of switch options on the introduction of PCV in Indonesia. Nevertheless, several limitations apply to this study. Firstly, in the absence of country-specific data available on wastage rate and cost of cold chain, our estimation was based on global data according to Gavi’s report [24]. Due to a lack of this data, we were unable to identify the cause of wastage and further analyze full costs of cold chain. However, if most of the wastage occurred due to cold chain failures like inadequate infrastructure, power shortages and poor maintenance, changing the vial size would not reduce this wastage [66]. To deal with this limitation, we took into account these parameters in sensitivity analysis. Secondly, due to the lack of data on the immunogenicity, safety and effectiveness of PCV10 B as the newest vaccine, part of our analysis on these issues was focused on PCV13 and PCV10 A. Another limitation is that this study did not consider the cost-effectiveness values of all PCVs that were compared. The cost-effectiveness values of PCVs are associated with many factors, including the burden of disease, vaccine effectiveness, indirect effects, vaccination coverage, vaccine price, delivery costs and schedule [68,69]. Although our cost-consequence approach is sometimes considered less rigorous than other economic evaluations, it is at the same time more versatile and practical, being able to offer clear and simple information. Despite the inherent limitations discussed, the current work represents a preliminary initial overview of switch options on the introduction of PCV in Indonesia, which is relevant with the current situation since the government of Indonesia has committed to introducing PCV into its routine immunization program with support from Gavi [70]. 

Several previous studies have indicated that PCV10 and PCV13 confer a comparable impact in pneumococcal disease overall [71,72,73]. Both products exhibit effectiveness and impact on overall disease and carriage. Nevertheless, there is no clear preference on using one product over the other in most settings. Several benefits of vaccination may be offset by increased rates of disease caused by non-covered serotypes. In settings with high burden attributable to serotype 3, PCV13 may have an additional benefit over PCV10. Both vaccines have a good safety profile, with no serious side effects on the individuals vaccinated [71,72,73]. The impact of PCV10 is similar to that of PCV13 across different subgroups of age, gender, race and socioeconomic status [14]. Both vaccines exhibit comparable impact and effectiveness overall on clinical outcomes. However, in settings of high serotypes 6A and 19A burden, PCV13 and PCV10 B may lead to greater reductions than PCV10 A, as these serotypes are contained in PCV13 and PCV10 B, and cross-protection from serotypes in PCV10 did not appear to offer the same magnitude of benefit as those observed from using PCV13 and PCV 10 B. 

Next to PCVs, other vaccines (e.g., diphtheria and tetanus toxoid combination vaccines, hepatitis B, poliovirus, H. influenzae type b, measles-containing and rabies vaccines) are considered interchangeable when administered under recommended indications [74,75,76,77]. The biggest challenge on the interchangeability of PCVs is that their composition differs more than for other types of vaccines. Despite the fact that a lot of previous studies recommended the completion of schedules with the same vaccine in accordance with the manufacturers’ recommendations [2,8,9,78,79,80,81], there is a growing number of countries that have switched from PCV13 to PCV 10 or *vice versa*, guided by and based on epidemiological or programmatic considerations [13,14,15,16,17,82,83,84,85,86]. Several countries implemented a gradual switch, in which a child’s schedule is completed with the vaccine given at first dose, while other countries completed the series with the new vaccine regardless of doses have been given. A key point that can be learned from the experience of Quebec, Canada is the use of a mixed schedule, which was based on immunogenicity, protection against IPD, pneumonia and AOM, herd effects, safety, cost-effectiveness, acceptability, feasibility and compliance [87]. In the epidemiological context of Quebec, a schedule consisting of PCV10 only or a mixed PCV10/PCV13 schedule would be more cost-effective than a PCV13 only schedule [87]. In addition, the government considered the high acquisition cost of PCV13, the absence of conclusive evidence that one vaccine is superior in preventing all pneumococcal diseases [7,78,88], the fact that PCV10 confers cross-protection against serotypes 6A and 19A IPD [89,90,91,92], the limited protective effect of PCV13 on serotype 3 IPD [93,94,95,96,97], and the impact of pertussis vaccination during pregnancy on the immunogenicity of a PCV13 infant schedule [98].

In the next couple of years, more studies are required to evaluate the interchangeability of PCVs by taking into account effectiveness and impact against overall IPD as the main outcome from the perspective of health economics. It is important to better understand the links between immunogenicity parameters and protection in real-life settings to enable interpretation of the results obtained in clinical and economics studies. Even though the interchangeability data are still scarce, the available evidence does not point to any major issues in switching from one PCV to another in NIPs. From a programmatic point of view, this issue is crucial since it may enable decision-makers to more easily switch to the most appropriate vaccine for their epidemiological or programmatic environment. 

## 8. Conclusions

Our analysis suggests an economic advantage to switch from single-dose into a multi-dose presentation. In terms of potential savings, the option of single-dose PCV13 with the contract price in 2019–2020 that would be switched into multi-dose PCV10 B with Gavi-AMC price in 2021–2024 resulted in the highest potential saving, compared with other scenarios. Vaccination coverage, vaccine price, vaccine wastage and additional cost of the Gavi-AMC vaccine were considered to be the most influential parameter affecting the savings in all scenarios. Applying the effectiveness of PCV13 and PCV10 A on reducing the risk for IPD, potential averted incidence of IPD in children under one year of age during 2019–2024 would be 246,164 and 105,587 in both scenarios. Despite the result confirmed that PCV13 may provide an additional benefit, a more comprehensive economic evaluation study is required to investigate further the comparison of cost-effectiveness values among all PCVs in Indonesia.

## Figures and Tables

**Table 1:** Characteristics of pneumococcal conjugate vaccines (PCVs; PCV13, PCV10 A and PCV10 B).

Characteristics	PCV13		PCV10 A	PCV10 B	
	1 Dose/Vial	4 Dose/Vial	4 Dose/Vial	1 Dose/Vial	5 Dose/Vial
Serotypes	1, 3, 4, 5, 6A, 6B, 7F, 9V, 14, 18C, 19A, 19F, 23F		1, 4, 5, 6B, 7F, 9V, 14, 18C, 19F, 23F	1, 5, 6A, 6B, 7F, 9V, 14, 19A, 19F, 23F	
WHO Prequalification	2010	2016	2017	2020	2020
Price per dose	$3.30 */$20.83 **	$2.90 *	$3.05 *	$3.50 *	$2.00 *
Doses per fully immunized child	3	3	3	3	3
Indicative wastage rate	5%	8%	8%	5%	8%
Shelf-life	36 months at 2–8 ℃	36 months at 2–8 ℃	36 months at 2–8 ℃	36 months at 2–8 ℃	36 months at 2–8 ℃
Cold chain volume per dose ***	12.6 cm³	3.9 cm³	2.7 cm³	17.0 cm³	3.7 cm³
Cost of cold room (per cm³) ****	$0.0013	$0.0013	$0.0013	$0.0013	$0.0013
Handling open vials	N.A.	Opened vials may be kept for use in subsequent immunization sessions (up to 28 days from the withdrawals of the first injection if held at 2 to 8 ℃)	Opened vials may be kept for use in subsequent immunization sessions (up to 28 days from the withdrawals of the first injection if held at 2 to 8 ℃)	N.A.	Opened vials may be kept for use in subsequent immunization sessions (up to 28 days from the withdrawals of the first injection if held at 2 to 8 ℃)

N.A. = Not applicable; * Gavi-AMC price with procurement via UNICEF SD [25]; ** Government contract price of PCV13; *** According to Gavi’s latest report [24]; **** According to a study by Parmar et al. and WHO Performance, Quality and Safety (PQS) standards [26,27].

**Table 2:** Input parameters.

Parameter	Value	Reference
Vaccine price		
PCV13—1 dose/vial (Government contract price)	Base value = $20.83 (Min = $18.94; Max = $61.00)	[22]
PCV13—1 dose/vial (UNICEF price) *	Base value = $3.30 (Lower = $2.48; Upper = $4.13)	[61]
PCV13—4 doses/vial (UNICEF price) *	Base value = $2.90 (Lower = $2.18; Upper = $3.63)	[61]
PCV10 A—4 doses/vial (UNICEF price) *	Base value = $3.05 (Lower = $2.29; Upper = $3.81)	[61]
PCV10 B—5 doses/vial (UNICEF price) *	Base value = $2.00 (Lower = $1.50; Upper = $2.50)	[61]
Additional cost		
Total additional cost **	Base value = 23.0% (Min = 19.5%, Max = 38.0%)	
Vaccine wastage		
Single-dose/vial	Median = 5.0% (Min = 1%; Max = 10%)	[26]
Multi-dose/vial	Median = 7.0% (Min = 1%; Max = 27%)	[26]
Safety stock		
Total safety stock	Base value = 5% (Min = 0%; Max = 10%)	
Vaccine coverage		
National coverage	Base value = 90.8% (Min = 46%, Max = 100%)	[25]
Cost of cold room		
Cost per cm3 ***	Base value = $0.0013 (Min = $0.0005; Max = $0.0022)	[26,27]
IPD in children <1 years old		
Incidence rate (per 100,000 population)	2911 (Range = 2265–3622)	[62]
Risk reduction for IPD		
PCV13	Odds Ratio = 0.53 (95% CI; 0.36–0.78)	[30]
PCV10 A	Odds Ratio = 0.19 (95% CI; 0.11–0.33)	[30]
PCV10 B	N.A.	

N.A.: not applicable; * Lower and upper value: ± 25%; ** Base value (costs of forwarder, distribution, insurance and service fee), Min (costs of the forwarder, distribution and insurance) and Max (costs of tax, import duty, forwarder, distribution, insurance and service fee); *** Base value (cold room size: 10 m^3^), Min (cold room size: 40 m^3^) and Max (cold room size: 5 m^3^).

**Table 3:** Targeted area of PCV introduction in Indonesia. Yellow, green and blue are for district, province and national level, respectively.

2019	2020	2021	2022	2023	2024
District/Province	District/Province	District/Province	District/Province	District/Province	District/Province
NTB	NTB	NTB	NTB	NTB	Indonesia
Bangka Belitung	Bangka Belitung	Bangka Belitung	Bangka Belitung	Bangka Belitung	
Kota Bogor	Jawa Barat	Jawa Barat	Jawa Barat	Jawa Barat	
Kota Bekasi	Jawa Timur	Jawa Timur	Jawa Timur	Jawa Timur	
Kota Surabaya		DKI Jakarta	DKI Jakarta	DKI Jakarta	
Gresik		Banten	Banten	Banten	
Sidoarjo		DI Yogyakarta	DI Yogyakarta	DI Yogyakarta	
		Jawa Tengah	Jawa Tengah	Jawa Tengah	
		Lampung	Lampung	Lampung	
		Sumatera Selatan	Sumatera Selatan	Sumatera Selatan	
			Bengkulu	Bengkulu	
			Jambi	Jambi	
			Riau	Riau	
			Kepulauan Riau	Kepulauan Riau	
			Sumatera Barat	Sumatera Barat	
			Sumatera Utara	Sumatera Utara	
			DI Aceh	DI Aceh	
			Bali	Bali	
				Gorontalo	
				Sulawesi Utara	
				Sulawesi Barat	
				Sulawesi Tengah	
				Sulawesi Selatan	
				Sulawesi Tenggara	
				Kalimantan Utara	
				Kalimantan Barat	
				Kalimantan Tengah	
				Kalimantan Selatan	
				Kalimantan Timur	
District					
Province					
Nationwide					

**Table 4:** Alternative scenario of switch options on the introduction of PCV.

Scenario	Year	Vaccine	Presentation	Price
Base-case	2019–2023	PCV13	1 dose/vial	Contract and Gavi-AMC price *
	2024	PCV13	4 doses/vial	Gavi-AMC price
Multi-dose PCV13	2019–2020	PCV13	1 dose/vial	Contract price
	2021–2024	PCV13	4 doses/vial	Gavi-AMC price
Multi-dose PCV10 A	2019–2020	PCV13	1 dose/vial	Contract price
	2021–2024	PCV10 A	4 doses/vial	Gavi-AMC price
Multi-dose PCV10 B	2019–2020	PCV13	1 dose/vial	Contract price
	2021–2024	PCV10 B	5 doses/vial	Gavi-AMC price

* Government contract price: 2019–2020; Gavi-AMC price: 2021–2024.

**Table 5:** Total potential saving on vaccine and cold chain cost among all scenarios.

Cost	2019	2020	2021	2022	2023	2024	Total
Vaccine cost							
Base case	$24,916,689	$97,407,970	$35,218,736	$46,179,392	$54,966,739	$50,065,979	$308,755,504
Multi-dose PCV13	$24,916,689	$97,407,970	$30,949,799	$40,581,890	$48,304,104	$50,065,979	$292,226,430
Multi-dose PCV10 A	$24,916,689	$97,407,970	$32,550,650	$42,680,953	$50,802,592	$52,655,598	$301,014,452
Multi-dose PCV10 B	$24,916,689	$97,407,970	$21,344,689	$27,987,510	$33,313,175	$34,528,261	$239,498,294
Cold chain cost							
Base case	$19,590	$76,586	$142,124	$186,356	$221,817	$71,162	$717,636
Multi-dose PCV13	$19,590	$76,586	$43,991	$57,682	$68,658	$71,162	$337,669
Multi-dose PCV10 A	$19,590	$76,586	$30,079	$39,440	$46,945	$48,658	$261,299
Multi-dose PCV10 B	$19,590	$76,586	$41,359	$54,231	$64.550	$66,904	$323,220
Total saving							
Multi-dose PCV13	$0	$0	$4,367,071	$5,726,176	$6,815,794	$0	$16,909,042
Multi-dose PCV10 A	$0	$0	$2,780,131	$3,645,354	$4,339,018	−$2,567,15	$8,197,389
Multi-dose PCV10 B	$0	$0	$13,974,813	$18,324,007	$21,810,831	$15,541,975	$69,651,626

## References

[1] Hausdorff W.P., Feikin D.R., Klugman K.P. (2005). Epidemiological differences among pneumococcal serotypes. Lancet Infect Dis..

[2] WHO (2019). Pneumococcal conjugate vaccines in infants and children under 5 years of age: WHO position paper—February 2019. Wkly. Epidemiol. Rec..

[3] Maimaiti N., Ahmed Z., Isa Z.M., Ghazi H.F., Aljunid S. (2013). Clinical Burden of Invasive Pneumococcal Disease in Selected Developing Countries. Value Health Reg. Issues.

[4] Ministry of Health, Republic of Indonesia (2017). Indonesia Health Profile 2017.

[5] UNICEF One Child Dies of Pneumonia Every 39 Seconds, Agencies Warn. https://www.unicef.org/indonesia/press-releases/one-child-dies-pneumonia-every-39-seconds-agencies-warn.

[6] World Health Organization Treatment and Prevention of Pneumonia. https://apps.who.int/gb/ebwha/pdf_files/WHA63/A63_26-en.pdf.

[7] SAGE Current Status of PCV Use and WHO Recommendations. https://www.who.int/immunization/sage/meetings/2017/october/01_17_October_2017_Presentation_01_OBrien_SAGE_PCV.pdf.

[8] European Medicines Agency Synflorix—Summary of Product Characteristics. http://www.ema.europa.eu/docs/en_GB/document_library/EPAR_-_Product_Information/human/000973/WC500054346.pdf.

[9] European Medicines Agency Prevenar 13—Summary of Product Characteristics. http://www.ema.europa.eu/docs/en_GB/document_library/EPAR_-_Product_Information/human/001104/WC500057247.pdf.

[10] Poolman J., Frasch C., Nurkka A., Käyhty H., Biemans R., Schuerman L. (2011). Impact of the conjugation method on the immunogenicity of Streptococcus pneumoniae serotype 19F polysaccharide in conjugate vaccines. Clin. Vaccine Immunol..

[11] Guevara J.N., Borys D., DeAntonio R., Guzman-Holst A., Hoet B. (2019). Interchangeability between pneumococcal conjugate vaccines for pediatric use: A systematic literature review. Expert Rev. Vaccines.

[12] Deceuninck G., De Serres G., Boulianne N., Lefebvre B., De Wals P. (2015). Effectiveness of three pneumococcal conjugate vaccines to prevent invasive pneumococcal disease in Quebec, Canada. Vaccine.

[13] Diawara I., Zerouali K., Katfy K., Zaki B., Belabbes H., Najib J., Elmdaghri N. (2015). Invasive pneumococcal disease among children younger than 5 years of age before and after introduction of pneumococcal conjugate vaccine in Casablanca, Morocco. Int. J. Infect. Dis..

[14] Ciapponi A., Lee A., Bardach A., Glujovsky D., Rey-Ares L., Luisa Cafferata M., Valanzasca P., Garcia Marti S. (2016). Interchangeability between Pneumococcal Conjugate Vaccines: A Systematic Review and Meta-Analysis. Value Health Reg. Issues.

[15] Su W.-J., Lo H.-Y., Chang C.-H., Chang L.-Y., Chiu C.-H., Lee P.-I., Lu C.-Y., Hsieh Y.-C., Lai M.-S., Lin T.-Y. (2016). Effectiveness of pneumococcal conjugate vaccines of different valences against invasive pneumococcal disease among children in Taiwan: A nationwide study. Pediatr. Infect. Dis. J..

[16] Johansson Kostenniemi U., Palm J., Silfverdal S.-A. (2018). Reductions in otitis and other respiratory tract infections following childhood pneumococcal vaccination. Acta Paediatr..

[17] Gouvernement du Québec Advice and Prevention—Pneumococcal Conjugate Vaccine. http://sante.gouv.qc.ca/en/conseils-et-prevention/vaccin-conjugue-contre-le-pneumocoque/.

[18] Australian Government, Department of Health Invasive Pneumococcal Disease in Australia, 2011 and 2012. http://www.health.gov.au/internet/main/publishing.nsf/Content/cda-cdi4002k.html.

[19] Cohen O., Knoll M., O’Brien K., Ramarkrishnan M., Constenla D., Privor-Dumm L., Buss-Younkin J., Farrar J., Pilishvili T., Whitney C. Pneumococcal Conjugate Vaccine (PCV) Product Assessment. https://www.jhsph.edu/ivac/wp-content/uploads/2018/05/pcv-product-assessment-april-25-2017.pdf.

[20] Hadinegoro S.R., Prayitno A., Khoeri M.M., Djelantik I.G., Dewi N.E., Indriyani S.A., Muttaqin Z., Mudaliana S., Safari D. (2016). Nasopharyngeal carriage of *Streptococcus Pneumoniae* in healthy children under five years old in Central Lombok Regency, Indonesia. Southeast Asian J. Trop. Med. Public Health.

[21] Dunne E.M., Murad C., Sudigdoadi S., Fadlyana E., Tarigan R., Indriyani S.A.K., Pell C.L., Watts E., Satzke C., Hinds J. (2018). Carriage of Streptococcus pneumoniae, Haemophilus influenzae, Moraxella catarrhalis, and Staphylococcus aureus in Indonesian children: A cross-sectional study. PLoS ONE.

[22] Ministry of Health, Republic of Indonesia (2020). Comprehensive Multi Year Plan (cMYP) of National Immunization Program 2020–2024.

[23] Gavi Indonesia. https://www.gavi.org/programmes-impact/country-hub/south-east-asia/indonesia.

[24] Gavi Gavi-Supported Pneumococcal Conjugate Vaccines Profiles to Support Country Decision Making. https://www.gavi.org/sites/default/files/document/pcv-profilespdf.pdf.

[25] Ministry of Health, Republic of Indonesia (2018). Indonesia Health Profile 2018.

[26] Parmar D., Baruwa E.M., Zuber P., Kone S. (2010). Impact of wastage on single and multi-dose vaccine vials: Implications for introducing pneumococcal vaccines in developing countries. Hum. Vaccines.

[27] WHO Performance, Quality and Safety (PQS) 2007. www.who.int/immunization_standards/vaccine_quality/pqs_prequalified_devices/en/index.html.

[28] Choe Y.J., Blatt D.B., Lee H.J., Choi E.H. (2020). Associations between geographic region and immune response variations to pneumococcal conjugate vaccines in clinical trials: A systematic review and meta-analysis. Int. J. Infect. Dis..

[29] Ngocho J.S., Magoma B., Olomi G.A., Mahande M.J., Msuya S.E., de Jonge M.I., Mmbaga B.T. (2019). Effectiveness of pneumococcal conjugate vaccines against invasive pneumococcal disease among children under five years of age in Africa: A systematic review. PLoS ONE.

[30] Ewald H., Briel M., Vuichard D., Kreutle V., Zhydkov A., Gloy V. (2016). The Clinical Effectiveness of Pneumococcal Conjugate Vaccines: A Systematic Review and Meta-analysis of Randomized Controlled Trials. Dtsch. Arztebl. Int..

[31] Sings H.L., De Wals P., Gessner B.D., Isturiz R., Laferriere C., McLaughlin J.M., Pelton S., Schmitt H.J., Suaya J.A., Jodar L. (2019). Effectiveness of 13-Valent Pneumococcal Conjugate Vaccine Against Invasive Disease Caused by Serotype 3 in Children: A Systematic Review and Meta-analysis of Observational Studies. Clin. Infect. Dis..

[32] Alicino C., Paganino C., Orsi A., Astengo M., Trucchi C., Icardi G., Ansaldi F. (2017). The impact of 10-valent and 13-valent pneumococcal conjugate vaccines on hospitalization for pneumonia in children: A systematic review and meta-analysis. Vaccine.

[33] Immunization Economics Cost-Effectiveness and Budget Impact Analyses of PCV in Indonesia. http://immunizationeconomics.org/baselposter/suwantika.

[34] WHO (2012). Pneumococcal vaccines WHO position paper–2012–recommendations. Vaccine.

[35] WHO Sustainable Access to Vaccines in Middle-Income Countries (MICs): A Shared Partner Strategy. https://www.who.int/immunization/sage/meetings/2015/april/Cernuschi_MIC_Strategy_SAGE_Apr2015.pdf?ua=1.

[36] World Bank The World Bank in Middle Income Countries. https://www.worldbank.org/en/country/mic/overview.

[37] WHO Global Immunization Data 2015. http://www.who.int/immunization/newsroom/press/immunization_coverage_july_2016/en/.

[38] Gavi Pneumococcal Vaccine Support. http://www.gavi.org/support/nvs/pneumococcal/.

[39] Tricarico S., McNeil H.C., Cleary D.W. (2017). Pneumococcal conjugate vaccine implementation in middle-income countries. Pneumonia.

[40] CDC CDC Vaccine Price List. https://www.cdc.gov/vaccines/programs/vfc/awardees/vaccine-management/price-list/index.html.

[41] World Bank Data Bank. http://databank.worldbank.org/data/reports.aspx?Code=SP.POP.TOTL&id=af3ce82b&report_name=Popular_indicators&populartype=series&ispopular=y.

[42] Gavi Historic Partnership between Gavi and India to Save Millions of Lives. http://www.gavi.org/library/news/press-releases/2016/historic-partnership-between-gavi-and-india-to-save-millions-of-lives/.

[43] Hu J., Sun X., Huang Z., Wagner A.L., Carlson B., Yang J., Tang S., Li Y., Boulton M.L., Yuan Z. (2016). Streptococcus pneumoniae and Haemophilus influenzae type b carriage in Chinese children aged 12–18 months in Shanghai, China: A cross-sectional study. BMC Infect. Dis..

[44] Kaddar M., Schmitt S., Makinen M., Milstien J. (2013). Global support for new vaccine implementation in middle-income countries. Vaccine.

[45] Richardson A., Morris D.E., Clarke S.C. (2014). Vaccination in Southeast Asia–reducing meningitis, sepsis and pneumonia with new and existing vaccines. Vaccine.

[46] WHO An Approach to Middle Income Countries: Options and Potential Impacts. https://www.who.int/immunization/sage/2_An_Approach_Middle_Income_Countries_Full_Paper.pdf.

[47] WHO Advanced Market Commitments for Vaccines. https://www.who.int/immunization/newsroom/amcs/en/.

[48] Cernuschi T., Furrer E., Schwalbe N., Jones A., Berndt E.R., McAdams S. (2011). Advance market commitment for pneumococcal vaccines: Putting theory into practice. Bull. World Health Organ..

[49] Barder O., Kremer M., Levine R. Making Markets for Vaccines: Ideas to Action (Working Group Report). http://www.cgdev.org/section/initiatives/_archive/vaccinedevelopment/chapters.

[50] Gavi An Advance Market Commitment for Pneumococcal Vaccines (Joint Donor Statement). http://www.gavialliance.org/library/documents/amc/iwg-joint-donor-statement/.

[51] Gavi Advance Market Commitment for Pneumococcal Vaccines (Annual Report 1 April 2010–31 March 2011). http://www.gavialliance.org/library/gavi-documents/amc/2011-pneumococcal-amc-annual-report/.

[52] UNICEF The Advance Market Commitment for Pneumococcal Vaccine. http://www.unicef.org/supply/index_60990.html.

[53] Gavi Advance Market Commitment for Pneumococcal Vaccine. https://www.gavi.org/sites/default/files/document/2015-pneumococcal-amc-annual-reportpdf.pdf.

[54] IFFIm International Finance Facility for Immunisation. http://www.iffim.org/Funding-Gavi/Results/Pneumococcal-vaccine/.

[55] Gavi AVI Project Review. http://www.gavi.org/results/evaluations/avi-project-review/.

[56] WHO, UNICEF Ending Preventable Child Deaths from Pneumonia and Diarrhoea by 2025. https://apps.who.int/iris/bitstream/handle/10665/79200/9789241505239_eng.pdf;jsessionid=5B5EA4F6490A1D258DA1279A52BFA765?sequence=1.

[57] WHO Benefits of Immunization. http://www.who.int/immunization/programmes_systems/supply_chain/benefits_of_immunization/en/.

[58] WHO Global Vaccine Action Plan Secretariat Annual Report 2015. http://www.who.int/immunization/global_vaccine_action_plan/gvap_.

[59] (2015). PAHO. http://www.paho.org/HQ/index.php?option=com_content&view=article&id=1864%3A2014-paho-revolving-fund&catid=839%3Arevolving-fund&Itemid=4135&lang=ensecretariat_report_2015.pdf.

[60] PATH Pneumonia and Pneumococcus Vaccine Development. http://sites.path.org/vaccinedevelopment/pneumonia-and-pneumococcus/vaccine-development/.

[61] UNICEF Vaccine Price List. www.unicef.org/supply/files/Product_Menu_0507.pdf.

[62] O’Brien K.L., Wolfson L.J., Watt J.P., Henkle E., Deloria-Knoll M., McCall N., Lee E., Mulholland K., Levine O.S., Cherian T. (2009). Burden of disease caused by Streptococcus pneumoniae in children younger than 5 years: Global estimates. Lancet.

[63] UNICEF Handling Fees. https://www.unicef.org/supply/index_62330.html.

[64] WHO Principles and Considerations for Adding a Vaccine to a National Immunization Programme: From Decision to Implementation and Monitoring. https://apps.who.int/iris/bitstream/handle/10665/111548/9789241506892_eng.pdf?sequence=1.

[65] World Bank, Gavi AMC Pilot Proposal 2006. www.vaccineamc.org/files/AMCPilotProposal.pdf.

[66] Usuf E., Mackenzie G., Ceesay L., Sowe D., Kampmann B., Roca A. (2018). Vaccine wastage in the Gambia: A prospective observational study. BMC Public Health.

[67] WHO Vaccine Management Handbook (2017). How to Calculate Vaccine Volumes and Cold Chain Capacity Requirements.

[68] Chaiyakunapruk N., Somkrua R., Hutubessy R., Henao A.M., Hombach J., Melegaro A., Edmunds J.W., Beutels P. (2011). Cost effectiveness of pediatric pneumococcal conjugate vaccines: A comparative assessment of decision-making tools. BMC Med..

[69] Saokaew S., Rayanakorn A., Wu D.B., Chaiyakunapruk N. (2016). Cost effectiveness of pneumococcal vaccination in children in low-and middle-income countries: A systematic review. Pharmacoeconomics.

[70] Gavi Indonesia Set to Introduce PCV into Routine Immunisation Programme. https://www.gavi.org/news/media-room/indonesia-protect-four-million-children-year-against-pneumonia.

[71] Trück J., Jawad S., Goldblatt D., Roalfe L., Snape M.D., Voysey M., Pollard A.J. (2016). The antibody response following a booster with either a 10- or 13-valent pneumococcal conjugate vaccine in toddlers primed with a 13-valent pneumococcal conjugate vaccine in early infancy. Pediatr. Infect. Dis. J..

[72] Los Santos A.M., Rodríguez-Weber M.A., Sánchez-Márquez P., Carreño-Manjarrez R., Cervantes-Apolinar M.Y., Ruiz-Guiñazú J., Ortega-Barria E., Borys D. (2017). Immunogenicity of a 2+1 Infant Vaccination Series with 13-Valent Pneumococcal Conjugate Vaccine (PCV13) Followed by Pneumococcal Non-Typeable Haemophilus influenzae Protein D Conjugate Vaccine (PHiD-CV): A Randomized Trial Exploring Interchangeability of PCVs. Open Forum. Infect. Dis..

[73] Urbancikova I., Prymula R., Goldblatt D., Roalfe L., Prymulova K., Kosina P. (2017). Immunogenicity and safety of a booster dose of the 13-valent pneumococcal conjugate vaccine in children primed with the 10-valent or 13-valent pneu- mococcal conjugate vaccine in the Czech Republic and Slovakia. Vaccine.

[74] Dolhain J., Janssens W., Mesaros N., Hanssens L., Fierens F. (2018). Hexavalent vaccines: Increasing options for policy-makers and providers. A review of the data supporting interchangeability (substitution with vaccines containing fewer antigens) and mixed schedules from the same manufacturer. Expert Rev. Vaccines.

[75] Feldman S. (2001). Interchangeability of vaccines. Pediatr. Infect. Dis. J..

[76] WHO (2018). Rabies vaccines: WHO position paper—April 2018. Wkly. Epidemiol. Rec..

[77] WHO (2017). Measles vaccines: WHO position paper—April 2017. Wkly. Epidemiol. Rec..

[78] WHO Pneumococcal Conjugate Vaccine (PCV) Review of Impact Evidence (PRIME): Summary of Findings from Systematic Review. https://www.who.int/immunization/sage/meetings/2017/october/3_FULL_PRIME_REPORT_2017Sep26.pdf?ua=1.

[79] WHO (2012). Pneumococcal vaccines WHO position paper—2012. Wkly. Epidemiol. Rec..

[80] PAHO Final Report of the Technical Advisory Group on Vaccine-Preventable Diseases. https://www.paho.org/hq/dmdocuments/2011/vaccination-tag19-2011-FinalReport-Eng.pdf.

[81] WHO (2017). Meeting of the Strategic Advisory Group of Experts on immunization, October 2017—Conclusions and recommendations. Wkly. Epidemiol. Rec..

[82] Desmet S., Verhaegen J., Van Ranst M., Peetermans W., Lagrou K. (2018). Switch in a childhood pneumococcal vaccination programme from PCV13 to PCV10: A defendable approach?. Lancet Infect. Dis..

[83] Desmet S., Peetermans W., Lagrou K. (2018). Switch in childhood pneumococcal vaccine in Belgium. Lancet Infect. Dis..

[84] Izurieta P., Breuer T. (2018). Interpretation of the switch in a childhood pneumococcal vaccination programme from PCV13 to PCV10 in Belgium. Lancet Infect. Dis..

[85] Mrkvan T., Pelton S.I., Ruiz-Guinazu J., Palmu A.A., Borys D. (2018). Effectiveness and impact of the 10-valent pneumococcal conjugate vaccine, PHiD-CV: Review of clinical trials and post-marketing experience. Expert Rev. Vaccines.

[86] Tsaban G., Ben-Shimol S. (2017). Indirect (herd) protection, following pneumococcal conjugated vaccines introduction: A systematic review of the literature. Vaccine.

[87] Institut National de Santé Publique du Québec (Comité sur l’immu- nisation du Québec) Scientific Advisory on the Optimal Schedule for Childhood Immunization Against Pneumococcal Disease in Québec. https://www.inspq.qc.ca/sites/default/files/publications/2379_opinion_optimal_schedule_childhood_immunization_pneumococcal_disease.pdf.

[88] de Oliveira L.H., Camacho L.A., Coutinho E.S., Martinez-Silveira M.S., Carvalho A.F., Ruiz-Matus C., Toscano C.M. (2016). Impact and effectiveness of 10 and 13-valent pneumococcal conjugate vaccines on hospitalization and mortality in children aged less than 5 years in Latin American countries: A systematic review. PLoS ONE.

[89] Domingues C.M., Verani J.R., Montenegro Renoiner E.I., de Cunto Brandileone M.C., Flannery B., de Oliveira L.H., Santos J.B., de Moraes J.C., Brazilian Pneumococcal Conjugate Vaccine Effectiveness Study Group (2014). Effectiveness of ten-valent pneumococcal conjugate vaccine against invasive pneumococcal disease in Brazil: A matched case-control study. Lancet Respir. Med..

[90] Jokinen J., Rinta-Kokko H., Siira L., Palmu A.A., Virtanen M.J., Nohynek H., Virolainen-Julkunen A., Toropainen M., Nuorti J.P. (2015). Impact of ten-valent pneumococcal conjugate vaccination on invasive pneumococcal disease in Finnish children–a population-based study. PLoS ONE.

[91] Rinta-Kokko H., Palmu A.A., Auranen K., Pekka Nuorti J., Toropainen M., Siira L., Virtanen M.J., Nohynek H., Jokinen J. (2018). Long-term impact of 10-valent pneumococcal conjugate vaccination on invasive pneumococcal disease among children in Finland. Vaccine.

[92] Verani J.R., Domingues C.M., de Moraes J.C. (2015). Indirect cohort analysis of 10-valent pneumococcal conjugate vaccine effectiveness against vaccine-type and vaccine-related invasive pneumococcal disease. Vaccine.

[93] Andrews N.J., Waight P.A., Burbidge P., Pearce E., Roalfe L., Zancolli M., Slack M., Ladhani S.N., Miller E., Goldblatt D. (2014). Serotype-specific effectiveness and correlates of protection for the 13-valent pneumococcal conjugate vaccine: A post licensure indirect cohort study. Lancet Infect. Dis..

[94] Dominguez A., Ciruela P., Hernandez S., García-García J.J., Soldevila N., Izquierdo C., Moraga-Llop F., Díaz A., de Sevilla M.F., González-Peris S. (2017). Effectiveness of the 13-valent pneumococcal conjugate vaccine in preventing invasive pneumococcal disease in children aged 7-59 months. A matched case-control study. PLoS ONE.

[95] Moore M.R., Link-Gelles R., Schaffner W., Lynfield R., Holtzman C., Harrison L.H., Schaffner W., Zansky S.M., Rosen J.B., Reingold L. (2016). Effectiveness of 13-valent pneumococcal conjugate vaccine for prevention of invasive pneumococcal disease in children in the USA: A matched case-control study. Lancet Respir. Med..

[96] van der Linden M., Falkenhorst G., Perniciaro S., Fitzner C., Imohl M. (2016). Effectiveness of Pneumococcal Conjugate Vaccines (PCV7 and PCV13) against invasive pneumococcal disease among children under two years of age in Germany. PLoS ONE.

[97] Weinberger R., van der Linden M., Imohl M., von Kries R. (2016). Vaccine effectiveness of PCV13 in a 3+1 vaccination schedule. Vaccine.

[98] Ladhani S.N., Andrews N.J., Southern J., Jones C.E., Amirthalingam G., Waight P.A., England A., Matheson M., Bai X., Findlow H. (2015). Antibody responses after primary immunization in infants born to women receiving a pertussis- containing vaccine during pregnancy: Single arm observational study with a historical comparator. Clin. Infect. Dis..

